# ﻿Two new species and a new host record of Hyphomycetes associated with decaying wood in Yunnan Province, China

**DOI:** 10.3897/mycokeys.121.162535

**Published:** 2025-09-02

**Authors:** Qinfang Zhang, Yulin Ren, Kamran Habib, Changtao Lu, Lili Liu, Jichuan Kang, Xiangchun Shen, Chuangen Lin, Nalin N. Wijayawardene, Hind A. Al-Shwaiman, Qirui Li, Abdallah M. Elgorban

**Affiliations:** 1 State Key Laboratory of Discovery and Utilization of Functional Components in Traditional Chinese Medicine & School of Pharmaceutical Sciences, Guizhou Medical University, Guian New District, Guizhou 550004, China; 2 The High Efficacy Application of Natural Medicinal Resources Engineering Centre of Guizhou Province (The Key Laboratory of Optimal Utilization of Natural Medicine Resources), School of Pharmaceutical Sciences, Guizhou Medical University, Gui'an, Guizhou, 561113, China; 3 Department of Botany, Khushal Khan Khattak University, Karak, KP, 27200 Pakistan; 4 School of Biology and Engineering (School of Health Medicine and Modern Industry), Guizhou Medical University, University Town, Guian New District, Guizhou 550025, China; 5 Engineering and Research Centre for Southwest Bio-Pharmaceutical, Resources of National Education Ministry of China, Guizhou University, Guiyang, Guizhou, 550025, China; 6 School of Life Sciences, Guizhou Normal University, Guiyang, 550025, China; 7 Center for Yunnan Plateau Biological Resources Protection and Utilization, College of Biology and Food Engineering, Qujing Normal University, Qujing, Yunnan 655011, China; 8 Department of Botany and Microbiology, College of Science, King Saud University, P.O. 2455, Riyadh 11451, Saudi Arabia; 9 Center of Excellence in Biotechnology Research (CEBR), DSR, King Saud University, P.O. Box. 2454 Riyadh, Saudi Arabia

**Keywords:** 2 new taxa, asexual morph, phylogeny, Sporidesmiales, taxonomy

## Abstract

In recent years, there has been certain progress in the research on fungal diversity in Yunnan Province, China, particularly in aquatic habitats. This study introduces two new species, *Ellisembia yuxiense* and *Sporidesmium ailaoshanense*, as well as a new host record of *Sporidesmium tropicale* on *Pinus yunnanensis* from freshwater habitats in Yunnan Province. All taxa were identified by integrating morphological traits with phylogenetic analysis of combined LSU, ITS, and *rpb*2 DNA sequences. Comprehensive morpho-anatomical descriptions and detailed illustrations are provided to elucidate the characteristics of each taxon.

## ﻿Introduction

The family Sporidesmiaceae was initially erected by Fries (1849) but remained neglected mainly until the availability of molecular data and re-examination of its type genus, *Sporidesmium*. Su et al. (2016) resurrected Sporidesmiaceae to accommodate specific taxa with available molecular data and morphological characteristics similar to *Sporidesmium ehrenbergii*, the lectotype species of *Sporidesmium*. Members of this family are typically saprobic or mycoparasitic, commonly found inhabiting decaying wood and plant debris in both terrestrial and freshwater ecosystems (Su et al. 2016). *Sporidesmium* was the sole genus in the family until Subramanian (1992) segregated *Ellisembia* to accommodate species characterized by distoseptate conidia (pseudosepta) and proliferating conidiophores.

However, given the lack of phylogenetic support for the taxonomic significance of euseptate and distoseptate conidia among *sporidesmium*-like species, Su et al. (2016) proposed that *Ellisembia* should be considered a synonym of *Sporidesmium* sensu stricto. However, Delgado et al. (2024) reevaluated the group using fresh collections of the type species *Ellisembia coronata* from its German type locality and rejected the synonymy of *Ellisembia* under *Sporidesmium*. Their multigene analyses revealed that *Ellisembia coronata* forms a distinct monophyletic lineage within Sporidesmiaceae, clearly separated from *Sporidesmium*. By restricting *Ellisembia* to this lineage—characterized by distoseptate conidia and conidiophores with few or no percurrent extensions—the authors realigned the genus with the original concept from Subramanian (1992). Currently, Species Fungorum lists 69 species under *Ellisembia* and 186 species under *Sporidesmium* (accessed 24 July 2025).

Our recent studies on aquatic fungi from Southwestern China have revealed several novel taxa and new records, including two novel species (*Memnoniella chrysanthemi* and *Craspedodidymum hunanense*) and a new record (*Aquadictyospora clematidis*) (Liu et al. 2024; Habib et al. 2025). In this study, we introduce two new species within the Sporidesmiaceae—*Ellisembia yuxiense* and *Sporidesmium ailaoshanense*—and report a new host record for another *Sporidesmium* species. Through phylogenetic analysis, we confirm the placement of these new species within their respective generic clades, while detailed morphological comparisons distinguish them from similar taxa.

## ﻿Materials and methods

### ﻿Sample collection

Between August and October 2024, researchers collected specimens of decaying branches and logs submerged in streams and lakes from Ailaoshan National Nature Reserve and Wumengshan National Nature Reserve in Yunnan Province, China. These specimens were packed in sealed plastic bags, and collection in formation was recorded (Rathnayaka et al. 2024); they were then transported to the mycology laboratory at Guizhou Medical University. The specimens were examined in the laboratory. Specimens that did not produce spores were placed in a sealed storage container and subjected to humidified incubation at 24 °C. Sterile water was sprayed into the container daily to maintain humidity levels, and the specimens were observed for spore production. All specimens were deposited at the herbarium of Guizhou Medical University (GMBH) and the Herbarium of Cryptogams, Herbarium of Kunming Institute of Botany, Chinese Academy of Sciences (KUN-HKAS).

### ﻿Morphological characterization and isolation

Macroscopic characteristics were examined under an Olympus SZ61 stereomicroscope (Japan) and photographed with a Canon 700D digital camera (Canon, Tokyo, Japan). Samples were mounted in water for microscopic observation. Quantitative measurements focused on internal structures, including the diameter, height, color, and shape of the conidiomata. The length and width of conidiophores and conidia were precisely measured, with width consistently recorded at the broadest point to ensure accuracy and comparability. The Tarosoft® Image Frame Work (v0.9.7) program and Adobe Photoshop CS6 software (Adobe Systems, USA) were used for measuring and processing images. Single-spore isolation was performed following the method described by Liu et al. (2024). A small volume of sterile water was used to suspend the conidia, which were then thoroughly mixed and evenly spread onto water agar (WA) plates. After 10 to 24 hours of incubation, germinated conidia were examined using a stereomicroscope and transferred to potato dextrose agar (PDA) plates. All cultures were incubated at 24 °C.

### ﻿DNA extraction, polymerase chain reaction (PCR) amplification, and sequencing

Colonies were cultivated on potato dextrose agar (PDA) plates for 2 to 4 weeks until the hyphae fully colonized the medium or growth ceased. Fresh mycelia were then gently scraped using a sterile scalpel for DNA extraction. Total genomic DNA was isolated using the BIOMIGA Fungal Genomic DNA Extraction Kit (TAKARA RR047A, China), following the manufacturer’s instructions. PCR amplification targeted three regions: the internal transcribed spacer (ITS), the large subunit rDNA (LSU), and a partial fragment of the second-largest subunit of RNA polymerase II (*rpb2*). Primer pairs used were ITS5/ITS4 for ITS (White et al. 1990), LR0R/LR5 for LSU (White et al. 1990), and fRPB2-5f/fRPB2-7cR for *rpb2* (Liu et al. 1999; Sung et al. 2007; Voglmayr et al. 2016). PCR reactions were performed in a 25 µL mixture containing 9.5 µL of double-distilled water, 12.5 µL of PCR Master Mix, 1 µL of each primer, and 1 µL of template DNA. PCR products were verified by 1.5% agarose gel electrophoresis, stained with GoldenView, and sent to Sangon Biotech (China) for sequencing.

### ﻿Phylogenetic analyses

The reference sequences retrieved from open databases originated from recently published data and BLASTn results of closely matching sequences. Sequences were aligned using the MAFFT v.7.110 online program (Katoh et al. 2019) with default settings. The alignments were adjusted manually using BioEdit v.7.0.5.3 (Hall 1999) where necessary. Maximum likelihood (ML) analyses were performed using RAxML v.8.2.12 with the GTRGAMMA substitution model and 1,000 bootstrap replicates (Stamatakis 2014). Phylogenetic analyses were also performed for Bayesian inference in MrBayes v.3.2.2 (Ronquist et al. 2012) online. Markov chain Monte Carlo (MCMC) sampling in MrBayes v.3.2.2 (Ronquist et al. 2012) was used to determine posterior probabilities (PP). Six simultaneous Markov chains were run for 1,000,000 generations, and trees were sampled every 1,000^th^ generation. The first 25% of the trees were discarded as burn-ins. The remainder was used to calculate the posterior probabilities (PPs) for individual branches. The phylogenetic tree was visualized in FIGTREE v.1.4.3 (Rambaut 2012). All analyses were conducted on the CIPRES Science Gateway v3.3 web portal (Miller et al. 2010). All obtained sequences were deposited in GenBank (Table 1). The outgroups and loci used in phylogenetic analyses, tailored to specific families and genera—including ITS, LSU, and *rpb2*—were chosen based on published literature. The best-scoring RAxML tree is presented in the phylogenetic figures, and the phylogenetic placement of isolated strains is detailed in the notes section of each respective taxon.

**Table 1.** Taxa and corresponding GenBank accession numbers of sequences used in the phylogenetic analysis.

Name	Strain	Type	GenBank accession numbers			References
			ITS	LSU	rpb2	
*Ellisembia bambusicola*	HKUCC 3578	–	–	DQ408562	–	Shenoy et al. 2006
*Ellisembia calyptrata*	HKUCC 10821	T	–	DQ408564	DQ435085	Tian et al. 2024
*Ellisembia cryptomeriae*	UESTCC 23.0227	T	OR887405	OR887115	PP076811	Tian et al. 2024
*Ellisembia coronata*	CCF 6699	–	OR886656	OR886656	–	Delgado et al. 2024
*Ellisembia cangshanensis*	MFLUCC 15-0420	–	–	KU376273	–	Su et al. 2016
*Ellisembia minigelatinosa*	NN 47497	–	–	DQ408567	–	Shenoy et al. 2006
*Ellisembia pseudobambusae*	CCF 6709	–	OR886657	OR886657	PP349741	Tian et al. 2024
*Ellisembia* sp.	HKUCC 10558	–	–	DQ408565	DQ435088	Shenoy et al. 2006
*Ellisembia melaleucae*	CPC 32707	T	MH327817	NG_064550	–	Tian et al. 2024
*Ellisembia melaleucae*	CPC 32936	–	MH327818	MH327854	–	Tian et al. 2024
*Ellisembia spiraeae*	CBS 148298	T	NR_175218	NG_081327	OK651164	Tian et al. 2024
*Ellisembia spiraeae*	CPC 39766	–	OK664716	OK663755	–	Tian et al. 2024
*Ellisembia yuxiensis*	GMB5104	T	PV779542	PV779549	PV78284	This study
*Ellisembia yuxiensis*	GMB5108	–	PV779546	PV779552	PV782985	This study
*Sporidesmium ailaoshanensis*	GMB5103	T	PV779542	PV779548	PV782982	This study
*Sporidesmium ailaoshanensis*	GMB5107	–	PV779545	PV779551	PV782983	This study
*Sporidesmium appendiculatum*	MFLU 18-0981	T	MW286500	MW287774	–	Dong et al. 2021
*Sporidesmium aturbinatum*	DLUCC 1417	–	MZ420743	MZ420758	MZ442697	Tian et al. 2024
*Sporidesmium chiangmaiense*	MFLUCC 18-0999	–	MW286497	MW287771	–	Dong et al. 2021
*Sporidesmium dulongense*	MFLUCC 17-0116	T	MH795812	MH795817	MH801190	Tian et al. 2024
*Sporidesmium fluminicola*	MFLUCC 15-0346	–	–	KU376271	–	Su et al. 2016
*Sporidesmium lageniforme*	MFLU 18-1594	T	MK828640	MK849782	MN124533	Tian et al. 2024
*Sporidesmium lignicola*	DLUCC 1376	–	–	MK849783	–	Yang et al. 2023
*Sporidesmium lignicola*	KUMCC 15-0266	T	–	MK849784	–	Yang et al. 2023
*Sporidesmium luminicola*	MFLUCC 15-0346	–	–	KU376271	–	Yang et al. 2023
*Sporidesmium parvum*	HKUCC 10836	–	–	DQ408558	–	Su et al. 2016
*Sporidesmium pyriformatum*	MFLUCC 15-0620	T	KX710146	KX710141	MF135649	Tian et al. 2024
*Sporidesmium pyriformatum*	MFLUCC 15-0627	–	KX710148	KX710143	MF135650	Tian et al. 2024
*Sporidesmium submersum*	MFLUCC 15-0421	T	–	KU376272	–	Su et al. 2016
*Sporidesmium tetracoilum*	CBS 126412	–	MH864106	MH875566	–	Vu et al. 2019
*Sporidesmium tetracoilum*	PRC 4681	–	OU413153	OU413153	–	Vu et al. 2019
*Sporidesmium thailandense*	MFLUCC 15-0964	T	–	MF374370	MF370955	Tian et al. 2024
*Sporidesmium thailandense*	MFLUCC 15-0617	–	–	MF077561	–	Delgado et al. 2024
*Sporidesmium tropicale*	MFLU 17–0850	–	MF077551	MF077562	MF135646	Yang et al. 2018
*Sporidesmium tropicale*	DLUCC:1689	–	MZ420745	MZ420760	MZ442698	Bao et al. 2021
*Sporidesmium tropicale*	MFLUCC 17-0344	–	OL780513	OL782088	–	Senwanna et al. 2021
*Sporidesmium tropicale*	GMB5105	–	PV779544	PV779550	PV782986	This study
*Sporidesmium tropicale*	GMB5109	–	PV779547	PV779553	PV782987	This study
*Sporoschisma hemipsilum*	CBS 414.61	–	MH858104	MH869677	–	Delgado et al. 2024
*Sporoschisma hemipsilum*	KUMCC 15-0227	–	KX455866	KX455859	–	Delgado et al. 2024
*Sporoschisma hemipsilum*	MFLUCC 17-1712	–	MK828616	MK835816	–	Delgado et al. 2024
*Sporoschisma juvenile*	MFLUCC 18-1348	–	MK828619	MK835819	–	Delgado et al. 2024
*Tracylla aristata*	CBS 141404	–	OL654129	OL654186	–	Tian et al. 2024
*Tracylla aristata*	CPC 25500	–	KX306770	KX306795	–	Tian et al. 2024

Notes: –: no data available; T: type specimens or strains.

## ﻿Results

### ﻿Phylogenetic analyses

**Figure 1.**
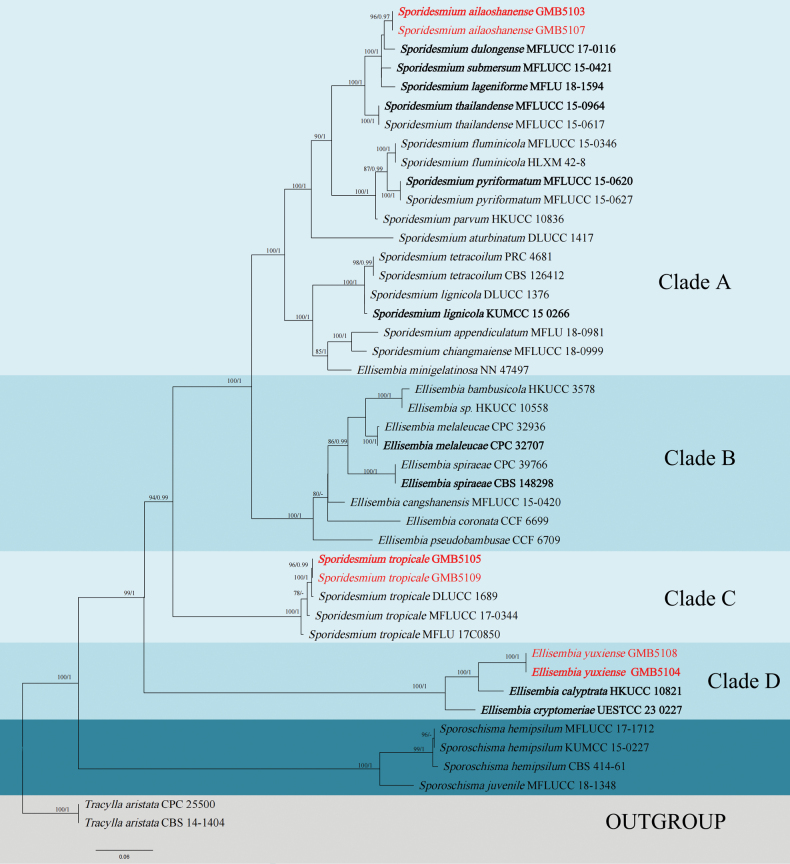
RAxML tree based on a combined ITS, LSU, and *rpb2* gene sequences data set. Bootstrap support values for maximum likelihood (ML) ≥ 75% and Bayesian posterior probabilities (BPP) ≥ 0.95 are displayed above or below the respective branches (ML/BI). New species and new host species are highlighted in red font, while type materials are displayed in bold black font.

The concatenated ITS–LSU–*rpb2* alignment of the Sporidesmiaceae dataset consisted of 46 taxa, including the outgroups. *Tracylla aristata* CBS-141404 and *T. aristata* CPC-25500 (Réblová et al. 2021) were selected as the outgroup taxon. The combined aligned sequence matrix comprises ITS (632 bp), LSU (1,568 bp), and *rpb2* (1,150 bp) sequence data, totaling 3,450 characters. The tree topology derived from maximum likelihood (ML) analysis closely resembled that of Bayesian inference (BI) analysis. The best-scoring RAxML tree is shown in Fig. 1. The phylogenetic tree based on BI and ML approaches confirmed the position of our newly generated sequences nested within the phylogenetic branch of the genera *Ellisembia* and *Sporidesmium* (Fig. 1). Two of our isolates cluster within the *Sporidesmium* clade, one representing a novel species that appeared as a sister to *Sporidesmium dulongense* (MFLUCC 17-0116), albeit with low statistical support. However, this topology was recovered consistently across multiple analyses. The second *Sporidesmium* isolate groups with *Sporidesmium tropicale* with high support (ML/BI = 100/1). The isolate of *Ellisembia yuxiense* sp. nov. (GMB5104, GMB5108) formed a well-supported clade (ML/BI = 100/1) that is sister to a lineage containing *E. calyptrata* (HKUCC 10821) and *E. cryptomeriae* (UESTCC 23 0227).

### ﻿Taxonomy

#### ﻿ Ellisembia yuxiense

Taxon classificationFungiHyphomycetesSporidesmiaceae

Q.F. Zhang, K. Habib & Q.R. Li sp. nov.

91ADC16D-B9B5-52F3-BB76-95EEDD4492C4

859647

Fig. 2

##### Etymology.

The specific epithet refers to the location where the holotype specimen was collected, Yuxi City.

##### Type.

China • Yunnan Province, Yuxi City, Ailaoshan National Nature Reserve (24°5'7.01"N, 101°31'30.44"E), altitude: 1169 m, on moist decayed branch, 15 September 2024, Qinfang Zhang, 2024ALS177-1 (GMB5104, holotype; GMBC5104, ex-type); KUN-HKAS 146987, isotype.

##### Description.

***Saprobic*** on decaying twigs of an unknown branch. ***Sexual morph***: undetermined. ***Asexual morph*: *Mycelium*** superficial, septate, light brown to brown, numerous, scattered, single or in groups. ***Conidiophores*** 81–158 × 4–6 µm (av. = 110.3 × 5.1 µm, *n* = 30), macronematous, mononematous, solitary or caespitose, erect, verruculose, straight or slightly curved, becoming slightly narrower towards the apex, 7–12-septate, smooth-walled, unbranched. ***Conidiogenous cells*** 3–6 × 2–4 µm (av. = 4.7 × 3.8 µm, *n* = 30), monoblastic, integrated, pale brown, terminal, cylindrical. ***Conidia*** 24–57 µm (av. = 40.4, *n* = 30) long, 6–9 µm (av. = 8.8 µm, *n* = 30) wide at the broadest part, tapering to 2–4 μm (av. = 3.4 μm, n = 30) wide at apex, 2–5 μm (av. = 3.4 μm, n = 30) wide at base, solitary, acrogenous, smooth, obclavate, truncate at the base, gray to light brown, without a mucilaginous cap, 5–9-distoseptate, and also 3–5 euseptate.

##### Culture characteristics.

Conidia germinate on water agar within 12 hours. At 24 °C, colonies growing on PDA reach a diameter of 10–15 mm after 7 weeks. Colonies convex, surface rough, moist, uneven, from above grayish-white, reverse dark brown to black. No pigmentation was produced in the culture medium.

**Figure 2.**
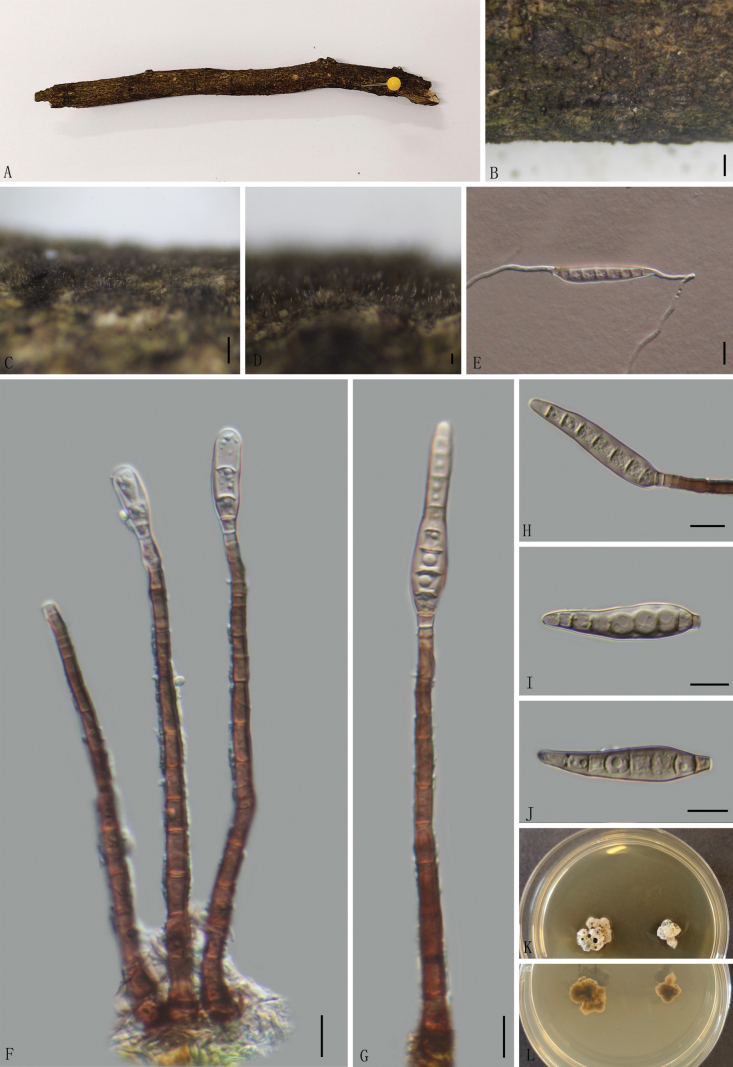
*Ellisembia yuxiense* (GMB5104, Holotype) **A.** Specimen; **B–D.** Conidiophores and conidia on natural substratum; **E.** Germinating conidium; **F, G.** Conidiophores and conidia; **H.** Conidiogenous cells and conidia; **I, J.** Conidia; **K, L.** Surface and reverse view of culture on PDA. Scale bars: 1 mm (**B**); 0.5 mm (**C**); 0.1 mm (**D**); 10 μm (**E–J**).

##### Additional material examined.

China • Yunnan Province, Yuxi City, Ailaoshan National Nature Reserve (28°19'21.77"N, 104°00'19.21"E), altitude: 1419 m, on moist decayed branch, 15 September 2024, Qinfang Zhang, 2024ALS175 (GMB5108; GMBC5108).

##### Notes.

Phylogenetically, *Ellisembia yuxiense* formed a well-supported sister branch to *E. calyptrata* (HKUCC-10821) (Fig. 1). However, the two species can be readily distinguished by conidiophore and conidia dimensions. *Ellisembia yuxiense* has significantly longer conidiophores (81–158 × 4–6 µm) compared to *E. calyptrata* (30–50 × 6–7 µm) and smaller conidia (24–57 × 6–9 µm vs. 60–90 × 9–12 µm) (Wu and Zhuang 2005; Shenoy et al. 2006). Morphologically, *Ellisembia yuxiense* resembles *E. cryptomeriae* in conidiophore size; however, the latter can be distinguished by its larger conidia, 20–85 × 7–14 µm with 6–17 septa, compared to *E. yuxiense*, which has conidia 24–57 × 6–9 µm with 5–9 septa. Differences from other morphologically similar species are provided in Table 2.

**Table 2.** Morphological comparison of Clade C (Fig. 1) and Ellisembia species.

Names	Strain	Conidiophores	Conidiogenous cells	Conidia	Reference
*Ellisembia calyptrata*	HKUCC 10821	verruculose. Brown to dark brown, 30–50 × 6.0–7.0 μm.	monoblastic, integrated, pale brown, terminal, cylindrical.	obclavate, truncate at the base, tapering to the apex, 6–17-septate, pale brown to dark brown, 60–90 × 9.0–12.0 μm.	Wu and Zhuang 2005
*E. coronata*	CCF 6699	(1–)2–5(–6)-septate, 0–2 ampulliform, lageniform or subcylindrical percurrent extensions, Brown to dark reddish brown, 18–66 × 5–7 μm.	monoblastic, integrated, pale brown, terminal, cylindrical	narrowly obclavate, less often subcylindrical or subfusiform, sometimes short rostrate, pale brown, smooth, 7–14-distoseptat, Brown to dark brown, (40–)52–89(–101) × 8–11 µm.	Delgado et al. 2024
*E. cryptomeriae*	UESTCC 23 0227	4–12-septate, verruculose. Brown to dark brown, 45–140 × 5–10 μm.	monoblastic, integrated, pale brown, terminal, cylindrical, 4–15 × 3–5 μm.	obclavate, truncate at the base, tapering to the apex, 6–17-septate, pale brown to dark brown, 20–85 × 7–14 μm.	Tian et al. 2024
*E. melaleucae*	CPC 32707	1-2-septate, erect, subcylindrical, dark brown, 12–30 × 4–6 μm.	terminal, medium brown, smooth, subcylindrical, holoblastic, 5–20 × 4–5 μm.	medium brown, smooth, obclavate, straight to flexuous, apexobtuse, base obconically truncate, 5-21-disto-septate, (45–)80–130(–170) × (8–)9–10(–11) μm.	Crous et al. 2018
*E. spiraeae*	CBS148298	1-4-septate, erect, dark brown, subcylindrical, unbranched, 15–40 × 5–7 μm.	terminal, integrated, subcylindrical, blastic, medium brown, smooth-walled, 10–20 × 5-–6 μm.	solitary, obclavate, straight to flexuous, apex subobtuse, base obconically truncate, (4-)6–10-distoseptate, (45–)70–85(–100) × (8–)9(–10) μm.	Crous et al. 2021
*E. yuxiense*	GMB5104, GMB5108	7–12-septate, verruculose. Brown to dark brown, 81–158 × 4–6 µm.	monoblastic, integrated, pale brown, terminal, cylindrical. 3–6 × 2–4 µm.	obclavate, truncate at the base, tapering to the apex, 5–9-septate, pale brown to dark brown. 24–57 × 6–9 µm.	This study

Furthermore, only the LSU and *rpb*2 sequence data are accessible for *E. calyptrata*. A comparison of sequence data of the LSU and *rpb*2 between *E. yuxiense* (GMB5104) and *E. calyptrata* (HKUCC-10821) shows 98.57% sequence identity in LSU and 88.72% sequence identity in *rpb*2.

#### ﻿ Sporidesmium ailaoshanense

Taxon classificationFungiHyphomycetesSporidesmiaceae

Q.F. Zhang & Q.R. Li sp. nov.

D8B45BDB-233D-503F-8E11-20EBD9DB86D8

859648

Fig. 3

##### Etymology.

The specific epithet refers to the location where the holotype specimen was collected, Ailaoshan National Nature Reserve.

##### Type.

China • Yunnan Province, Ailaoshan National Nature Reserve (24°5'7.01"N, 101°31'30.44"E), altitude: 1169 m, on dry rotten wood, 15 September 2024, Qinfang Zhang, 2024ALS74 (GMB5103, holotype; GMBC5103, ex-type); KUN-HKAS 146988, isotype.

##### Description.

***Saprobic*** on decaying twigs of an unknown wood. ***Sexual morph***: undetermined. ***Asexual morph*: *Colonies*** on the substratum superficial, effuse, scattered, hairy, black. ***Mycelium*** immersed, composed of branched, septate, smooth, pale brown to brown hyphae. ***Conidiophores*** 110–184 × 4–7 µm (av. = 135 × 6.2 µm, *n* = 30), macronematous, mononematous, unbranched, erect, straight or slightly flexuous, smooth, thick-walled, septate, not clear, cylindrical, dark brown, paler towards apex, smooth, and thick-walled. ***Conidiogenous cells*** 10–16 × 4–9 µm (av. = 13.2 × 7.1 µm, *n* = 30), monoblastic, integrated, determinate or sometimes percurrently proliferating, terminal, pale brown, cylindrical. ***Conidia*** 42–58 × 13–22 µm (av. = 49.0 × 17.4 µm, *n* = 30), acrogenous, solitary, dry, pyriform or lageniform, truncate at the base, smooth, dark brown, paler towards the apex, 4–5 µm wide and truncate at the base, thick-walled, 3–5 µm wide at the apex, 6–8-septate, slightly constricted at the septa, ***Conidial secession*** schizolytic.

**Figure 3.**
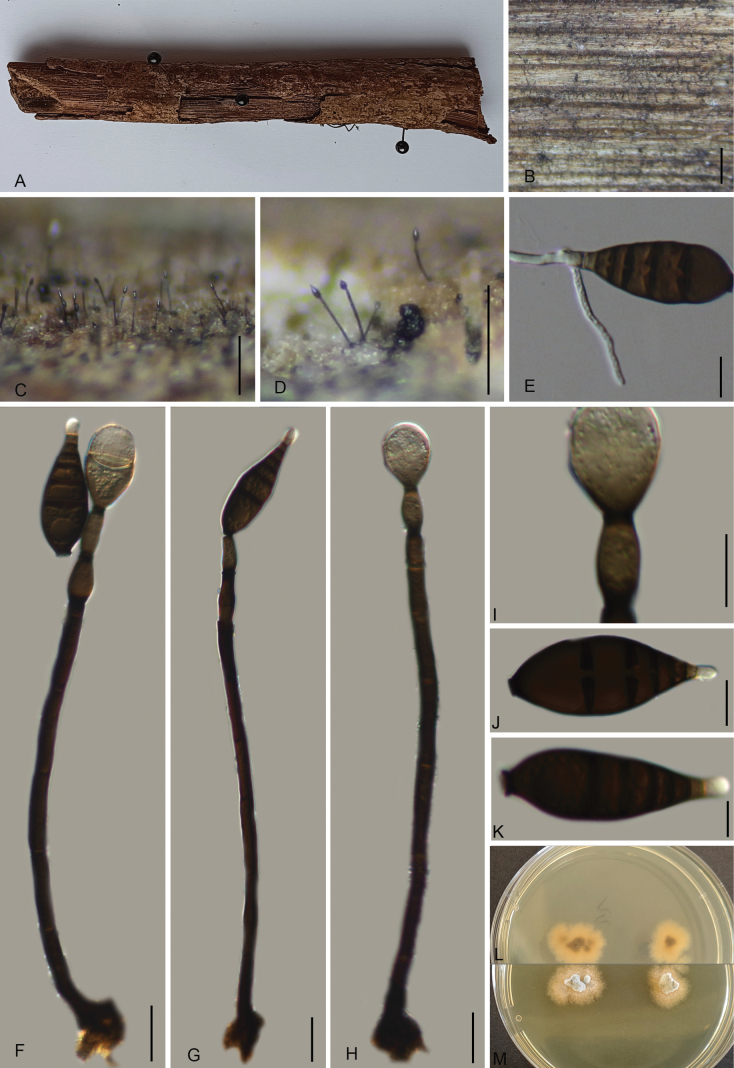
Sporidesmium ailaoshanense (GMB5103, Holotype) **A.** Specimen; **B–D.** Conidiophores and conidia on natural substratum; **E.** Germinating conidium; **F–H.** Conidiophores and conidia; **I.** Conidiogenous cell; **J, K.** Conidia; **L, M.** Surface and reverse view of culture on PDA. Scale bars: 1 mm (**B**); 0.25 mm (**C, D**); 10 μm (**E–K**).

##### Culture characteristics.

Conidia germinate on WA within 12 hours. At 25 °C, colonies growing on PDA reach a diameter of 20–30 mm after three weeks. The colonies are convex, with a smooth surface, mycelium present, dry, flat, and wrinkle-free. From above, the center appears white, with a grayish-white edge, while from below, the colony center is dark brown to black. No pigmentation is produced in the culture medium.

##### Additional material examined.

China • Yunnan Province, Ailaoshan National Nature Reserve (24°5'4.82"N, 101°31'32.89"E), altitude: 1131 m, on a dry wood branch, 15 September 2024, Qinfang Zhang, 2024ALS131 (GMB5107, GMBC5107).

##### Notes.

Phylogenetically, *Sporidesmium ailaoshanense* is closely related to *S. dulongense* (MFLUCC-17-0116) (Fig. 1). Morphologically, the two species share similar conidial shape and length. However, *S. ailaoshanense* can be distinguished by its longer conidiophores (110–184 μm vs. 88–124 μm) and wider conidia (13–22 μm vs. 13–15 μm) (Hyde et al. 2020). Moreover, *S. dulongense* has conidia with a long hyaline apex, which is very short in *S. ailaoshanense.* Base pair comparisons also support their separation; the ITS, LSU, and *rpb*2 sequences of *S. ailaoshanense* (GMB5103) and *S. dulongense* (MFLUCC-17-0116) show 97.0% sequence identity in ITS, 99.6% in LSU, and 97.64% in *rpb*2.

*Sporidesmium submersum* also shares a similar conidial shape and length with *S. ailaoshanense*, including a short hyaline apex. However, it differs in having much shorter conidiophores (59–72 μm vs. 110–184 μm) and thinner conidia (14–16 μm vs. 13–22 μm) (Su et al. 2016).

#### ﻿ Sporidesmium tropicale

Taxon classificationFungiHyphomycetesSporidesmiaceae

M.B. Ellis, Mycol. Pap. 70: 58 (1958)

36AB9057-A791-5431-A929-E083BD4E830C

306326

Fig. 4

##### Host.

*Pinus yunnanensis* Franch.

##### Description.

***Saprobic*** on submerged decaying branch of *Pinus yunnanensis*. ***Sexual morph***: undetermined. ***Asexual morph*: *Colonies*** on superficial substratum, scattered, hairy, effuse, and black. ***Mycelium*** mostly immersed, composed of septate, branched, pale black, and smooth-walled hyphae. ***Conidiophores*** 76–392 × 4–8 µm (av. = 239 × 7.0 µm, *n* = 30), macronematous, mononematous, unbranched, cylindrical, erect, straight or slightly flexuous, single, 5–17-septate, dark brown, paler towards apex, smooth, and thick-walled. ***Conidiogenous cells*** 4–11 × 3–5 µm (av. = 6.1 × 4.0 µm, *n* = 30), monoblastic, holoblastic, terminal, integrated, cylindrical, and dark-brown. ***Conidia*** 65–134 × 12–16 µm (av. = 105 × 14.8 µm, *n* = 30), acrogenous, solitary, dry, pyriform, rostrate, obclavate, with a long and slender apex, straight or slightly curved, tapering to the apex, 3–5 µm wide and truncate at the base, dark brown, pale brown, 2–5 µm wide at the apex, 11–17-septate, thick-walled, and with the proximal part usually verrucose.

##### Material examined.

China • Yunnan Province, Wumengshan National Nature Reserve (28°19'29.79"N, 104°00'05.48"E), altitude: 1361 m, on *Pinus yunnanensis* Franch., decaying wood, 22 July 2024, Qinfang Zhang, 2024WMS80 (GMB5105). China • Yunnan Province, Wumengshan National Nature Reserve (28°19'15.23"N, 104°0'16.31"E), altitude: 1373 m, on dry wood, 22 July 2024, Qinfang Zhang, 2024WMS96 (GMB5109).

##### Notes.

In the phylogram (Fig. 1), our collections (*Sporidesmium tropicale* GMB5105 and GMB5109) clustered with *S. tropicale* (DLUCC-1689) with strong statistical support (ML/BY: 100/1). DNA sequence comparisons revealed high similarity to *S. tropicale* (DLUCC-1689), with 99.38% identity in the ITS, 100.00% in the LSU, and 99.71% in the *rpb*2 gene. However, noticeable morphological differences were observed when compared to both the original description by Ellis (1958) and the description provided by Bao et al. (2021), particularly in the size of the conidiophores and conidia.

The conidiophores in our collection (*S. tropicale* GMB5105) are longer (76–392 × 4–8 µm) than those reported by Bao et al. (2021) for *S. tropicale* (DLUCC-1689) (71–163 × 5–8 µm), while the conidia are slightly smaller (65–134 × 12–16 µm vs. 94–184 × 13–15 µm). In the original description by Ellis (1958), *S. tropicale* was characterized by even longer conidiophores (40–340 µm) and larger conidia (80–230 µm), highlighting the morphological variability. These differences may be attributed to environmental factors such as geography, humidity, temperature, or the developmental stage of the specimens. However, the morphological characteristics of our specimens fall within the range of the features described in the original description by Ellis (1958). Previously, *S. tropicale* has been reported on dead branches of various dicotyledonous plants, including *Averrhoa carambola*, *Blighia unijugata*, *Camellia sinensis*, *Psidium guajava*, *Chrysophyllum albidum*, *Cola nitida*, *Dillenia indica*, *Hevea brasiliensis*, *Glyphaea brevis*, *Homalium aylmeri*, *Lecaniodiscus cupanioides*, *Ochthocosmus africanus*, *Parinari excelsa*, *Phyllanthus discoideus*, and *Pimenta officinalis*. In this study, we report *S. tropicale* on a new host monocot plant, *Pinus yunnanensis*. The strain GMB5105 is therefore designated as a new host record.

**Figure 4.**
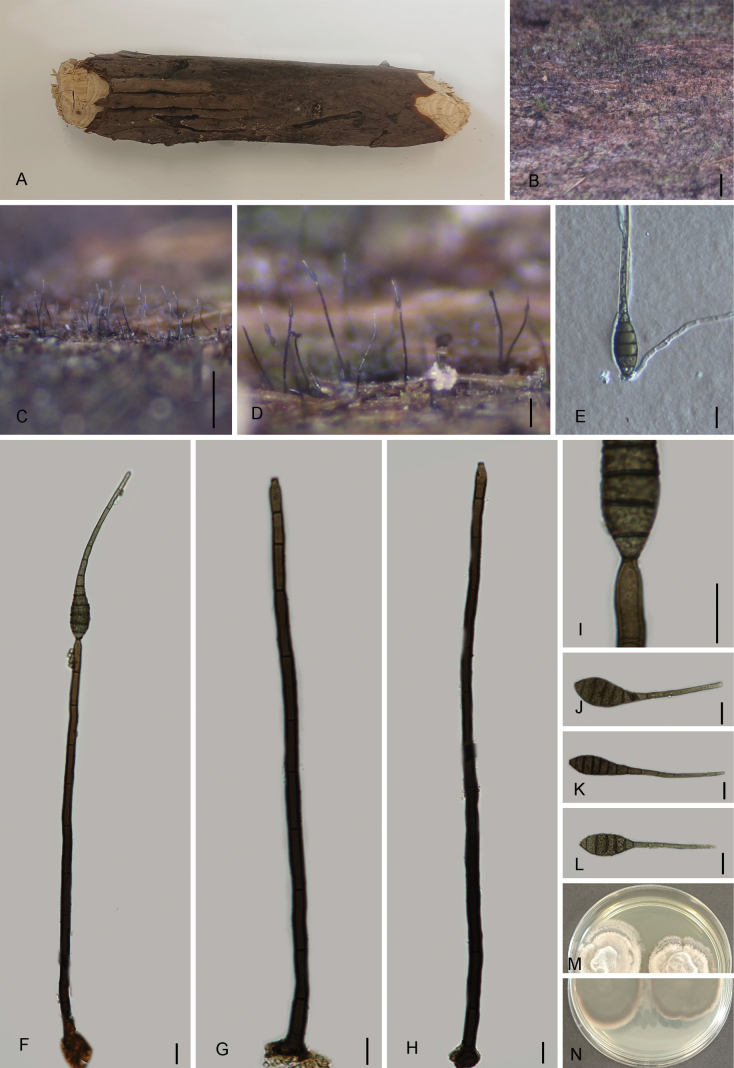
*Sporidesmium tropicale* (GMB5109) **A.** Specimen; **B–D.** Conidiophores and conidia on natural substratum; **E.** Germinating conidium; **F–H.** Conidiophores and Conidia; **I.** Conidiogenous cells; **J, M.** Conidia. Scale bars: 1 mm (**B**); 0.5 mm (**C, D**); 10 μm (**E–M**).

## ﻿Discussion

### ﻿Analysis and discussion of *Sporidesmium tropicale* in Clade C

In our phylogenetic analysis (Fig. 1), Clade C encompasses *Sporidesmium tropicale* (GMB5105, GMB5109), *S. tropicale* (DLUCC 1689), *S. tropicale* (MFLUCC 17–0344), and *S. tropicale* (MFLU 17C0850). It forms an independent branch distinct from Clade A (*Sporidesmium*) and Clade B (*Ellisembia*) with high support values (ML/BI = 94/0.99). Morphologically, the conidiophores of *S. tropicale* are relatively similar to those of *Sporidesmium* species. However, there are notable differences in their conidia. The conidia of *S. tropicale* are pyriform, rostrate, and obclavate, with a long and slender apex. They are straight or slightly curved, tapering toward the apex. Their color is dark brown, with a paler brown apex that is 2–3 µm wide; they possess 4–17 septa. In contrast, the conidia of *sporidesmium*-like species (Clade A) are obclavate or cylindrical with tapering ends, a hyaline apex, and a color ranging from pale brown to brown. Additionally, the conidiogenous cells of *S. tropicale* are holoblastic, whereas those of *sporidesmium*-like species (Clade A) are percurrent (Table 2) (Zhang et al. 2017; Yang et al. 2018; Bao et al. 2021; Senwanna et al. 2021).

Based on the morphological differences between *S. tropicale* and *Sporidesmium* species, this study suggests that *S. tropicale* may be classified as a distinct genus. However, to date, no similar species have been reported. Consequently, the criteria for establishing it as a separate genus have not been met, and the original species name is retained in this study.

### ﻿Morphological discussion and analysis of the three species in Clade D

In our phylogenetic analysis (Fig. 1), Clade D encompasses *Ellisembia yuxiense* (GMB5104, GMB5108), *Ellisembia calyptrata* (HKUCC 10821), and *Ellisembia cryptomeriae* (UESTCC 23 0227). It forms an independent branch distinct from Clade A, Clade B, and Clade C (Sporidesmiaceae) with high statistical values (ML/BI = 99/1). Morphologically, notable size disparities are observed in both conidiophores and among *E. yuxiense* (GMB5104, GMB5108), *E. calyptrata* (HKUCC 10821), and *E. cryptomeriae* (UESTCC 23 0227) (Wu and Zhuang 2005; Tian et al. 2024). Furthermore, the conidiogenous cells of *E. yuxiense* (GMB5104, GMB5108) exhibit similarities to those of the type species of *Ellisembia*: *Ellisembia coronata* (CCF 6699). However, the differences between these two species are primarily manifested in the variations in size and color of their conidiophores and conidia (Table 2).

### ﻿Discovery of new fungal species and host records enriches understanding of Sporidesmiaceae diversity and ecology in Yunnan Province, China

The discovery of two new fungal species (*E. yuxiense* and *S. ailaoshanense*) and a new host record (*S. tropicale* on *Pinus yunnanensis*) enhances our understanding of fungal diversity in Yunnan, China. Integrating morphological and molecular data is crucial for accurate classification in Sporidesmiaceae. Phylogenetic analyses support these new taxa and clarify their relationships. Morphological differences align with molecular divergences, highlighting ecological adaptability and evolutionary divergence. The new host record expands *S. tropicale*’s ecological range, suggesting host-specific adaptations. Further studies are needed to investigate their ecological roles and distribution patterns (Hyde et al. 2020; Bao et al. 2021).

## ﻿Conclusion

Our study highlights the distinct evolutionary positions of *Sporidesmium tropicale* in Clade B and *Ellisembia* species in Clade C within Sporidesmiaceae, supported by high phylogenetic support values. Morphological disparities, particularly in conidial characteristics and conidiogenous cell types, underscore potential taxonomic distinctions.

However, there is currently insufficient evidence to warrant the establishment of new genera. The discovery of new species and a host record enhances our understanding of fungal diversity in Yunnan, emphasizing the need for integrated morphological and molecular approaches in future taxonomic and ecological research.

## Supplementary Material

XML Treatment for Ellisembia yuxiense

XML Treatment for Sporidesmium ailaoshanense

XML Treatment for Sporidesmium tropicale

## ﻿Additional information

### Use of AI

No use of AI was reported.

### Author ORCIDs

Qinfang Zhang https://orcid.org/0009-0003-9408-4988

Yulin Ren https://orcid.org/0009-0003-9063-425X

Kamran Habib https://orcid.org/0000-0003-2572-0306

Jichuan Kang https://orcid.org/0000-0002-6294-5793

Xiangchun Shen https://orcid.org/0000-0002-4333-9106

Chuangen Lin https://orcid.org/0000-0003-2750-8720

Nalin N. Wijayawardene https://orcid.org/0000-0003-0522-5498

Abdallah M. Elgorban https://orcid.org/0000-0003-3664-7853

Qirui Li https://orcid.org/0000-0001-8735-2890

### Data availability

All specimens are deposited in the herbaria of Guizhou Medical University (GMBH) and the Kunming Institute of Botany, Chinese Academy of Sciences (KUN-HKAS). Sequences have been deposited in GenBank. The alignment file can be obtained from the corresponding author upon request.
